# Monocyte Chemoattractant Protein-1 Is an Independent Predictor of Coronary Artery Ectasia in Patients with Acute Coronary Syndrome

**DOI:** 10.3390/jcm9093037

**Published:** 2020-09-21

**Authors:** Juan Antonio Franco-Peláez, Roberto Martín-Reyes, Ana María Pello-Lázaro, Álvaro Aceña, Óscar Lorenzo, José Luis Martín-Ventura, Luis Blanco-Colio, María Luisa González-Casaus, Ignacio Hernández-González, Rocío Carda, María Luisa Martín-Mariscal, Jesús Egido, José Tuñón

**Affiliations:** 1 Department of Cardiology, Instituto de Investigación Sanitaria-Fundación Jiménez Díaz, 28040 Madrid, Spain; ampello@quironsalud.es (A.M.P.-L.); aacena@quironsalud.es (Á.A.); rcarda@quironsalud.es (R.C.); jtunon@fjd.es (J.T.); 2 Department of Medicine, Autónoma University, 28049 Madrid, Spain; olorenzo@quironsalud.es (Ó.L.); JLMartin@fjd.es (J.L.M.-V.); jegido@fjd.es (J.E.); 3 Department of Cardiology, Hospital La Luz, 28003 Madrid, Spain; rmartinr@quironsalud.es; 4 Laboratory of Vascular Pathology, Instituto de Investigación Sanitaria-Fundación Jiménez Díaz, 28040 Madrid, Spain; lblanco@fjd.es; 5 Centro de Investigación Biomédica en Red (CiberCV), 28029 Madrid, Spain; 6 Centro de Investigación Biomédica en Red de Diabetes y Enfermedades Metabólicas Asociadas (CIBERDEM), 28029 Madrid, Spain; 7 Laboratory of Nephrology and Mineral Metabolism, Hospital Gómez-Ulla, 28047 Madrid, Spain; mlgcasaus@gmail.com; 8 Department of Cardiology, Hospital Universitario Río Hortega, 47012 Valladolid, Spain; hgnacho@hotmail.com; 9 Department of Cardiology, Hospital Ruber Juan Bravo, 28006 Madrid, Spain; luisa.mmariscal@quironsalud.es; 10 Department of Nephrology, Instituto de Investigación Sanitaria-Fundación Jiménez Díaz, 28040 Madrid, Spain

**Keywords:** coronary artery ectasia, monocyte chemoattractant protein-1, low-density lipoprotein, acute atherotrombotic events, coronary artery disease

## Abstract

Our purpose was to assess a possible association of inflammatory, lipid and mineral metabolism biomarkers with coronary artery ectasia (CAE) and to determine a possible association of this with acute atherotrombotic events (AAT). We studied 270 patients who underwent coronary angiography during an acute coronary syndrome 6 months before. Plasma levels of several biomarkers were assessed, and patients were followed during a median of 5.35 (3.88–6.65) years. Two interventional cardiologists reviewed the coronary angiograms, diagnosing CAE according to previously published criteria in 23 patients (8.5%). Multivariate binary logistic regression analysis was used to search for independent predictors of CAE. Multivariate analysis revealed that, aside from gender and a diagnosis of dyslipidemia, only monocyte chemoattractant protein-1 (MCP-1) (OR = 2.25, 95%CI = (1.35–3.76) for each increase of 100 pg/mL, *p* = 0.001) was independent predictor of CAE, whereas mineral metabolism markers or proprotein convertase subtilisin/kexin type 9 were not. Moreover, CAE was a strong predictor of AAT during follow-up after adjustment for other clinically relevant variables (HR = 2.67, 95%CI = (1.22–5.82), *p* = 0.013). This is the first report showing that MCP-1 is an independent predictor of CAE, suggesting that CAE and coronary artery disease may share pathogenic mechanisms. Furthermore, CAE was associated with an increased incidence of AAT.

## 1. Introduction

Coronary artery ectasia (CAE) is a characteristic disorder of the coronary arteries, with a prevalence of 0.2–5% in patients undergoing cardiac catheterization [1,2,3,4]. Angiographic findings suggestive of CAE comprise segmental or diffuse dilatation of one or several coronary arteries, with slow and turbulent flow. Clinically, patients diagnosed with CAE are prone to exercise-induced angina and acute atherotrombotic events (AAE) regardless of the degree of stenotic coronary artery disease (CAD). Classically, data about natural history have been scarce and even contradictory [3,5].

Little is known about the pathophysiology of CAE. It has been related to atherosclerosis, congenital disorders, Kawasaki disease, and aneurysms in other territories, although it is difficult to differentiate among these types in the clinical setting [6,7,8,9]. Classical studies showed similar histological changes to those of severe atherosclerosis, although other patients presented smooth muscle hyalinization of the coronary media layer [6,10]. Moreover, inflammation could play a role in CAE development, as these patients present higher plasmatic levels of cytokines (interleukin-6 and tumor necrosis factor), inflammatory cells (leucocytes, monocytes), high sensitivity C-reactive protein (hs-CRP), soluble adhesion molecules, and neutrophil gelatinase-associated lipocalin (NGAL) [11,12,13,14,15,16].

Monocyte chemoattractant protein-1 (MCP-1) is one of the most important cytokines related to the pathogenesis of atherosclerosis, which plays a main role in the recruitment of monocytes into the vascular wall [17,18]. Furthermore, MCP-1 blood levels have been associated with increased incidence of acute ischemic events in patients with CAD [19]. As far as we know, a possible relationship of MCP-1 with CAE has not been studied.

In recent years, mineral metabolism components have been related to atherosclerosis [20]. In addition, an independent association between CAE and vitamin D deficiency has been found [21]. However, a possible relationship between CAE and other components of mineral metabolism (e.g., fibroblast growth factor-23 (FGF-23), parathormone and phosphate plasma levels) has not been previously investigated. 

On the other hand, there is an increasing interest in the role of proprotein convertase subtilisin/kexin type 9 (PCSK9) in the pathophysiology of atherosclerosis, but there are no data about its relationship with CAE.

We studied 270 patients with CAD in order to assess whether plasma levels of several biomarkers related to inflammation and lipid or mineral metabolism were predictors of the existence of CAE. Moreover, we aimed to assess if the presence of CAE is related with an increased risk of AAE.

## 2. Methods

### 2.1. Patients and Study Design

The present work is a sub-study of The Biomarkers in Acute Coronary Syndrome (BACS) and Biomarkers in Acute Myocardial Infarction (BAMI) studies that included patients with non-ST-elevation acute coronary syndrome (NSTEACS) or ST-elevation myocardial infarction (STEMI). Although the original study included patients admitted at 5 hospitals (Fundación Jiménez Díaz, Fuenlabrada, Móstoles, Puerta de Hierro and Alcorcón in Spain), in this sub-study, we have recruited only patients admitted at Fundación Jiménez Díaz. The design and inclusion/exclusion criteria of the BACS&BAMI studies have been published previously [19]. Briefly, patients with NSTEACS or STEMI were included during the index event. Exclusion criteria were age >85 years, other significant cardiac disorders except left ventricular hypertrophy secondary to hypertension, any illness or toxic habits that might limit patient survival, impossibility to perform revascularization when indicated, patients not stable at day six after the index event, and subjects in whom follow-up was not possible. Six to twelve months later, on an outpatient basis, blood was extracted isolating and freezing the plasma at −80 °C. Between July 2006 and April 2010, 676 patients were discharged from our Institution with a diagnosis of NSTEACS or STEMI and had undergone coronary angiography. Of these, 284 were included in the BACS&BAMI study, although in 14 patients the coronary angiogram was not available at the time of the review. Therefore, 270 patients were included in the final analysis. The remaining patients were not included due to the following: age over 85 years (16.3%), disorders or toxic habits limiting survival (35.8%), impossibility to carry out cardiac revascularization (13.5%), coexistence of other significant cardiopathy (5.8%), impossibility to perform follow-up (12.0%), clinical instability beyond the day 6 at the index event (10.1%), refusal to be included in the study (0.9%), death before the second visit (0.3%), and impossibility of the investigators to include them (5.3%). 

### 2.2. Coronary Angiogram Analysis

All patients underwent cardiac catheterization, and revascularization if indicated, at index admission. Two experienced interventional cardiologists (JAFP and RMR), who were unaware of clinical or analytical data, reviewed all coronary angiograms together. CAE was defined as a dilatation of an arterial segment to a diameter at least 1.5 times that of the adjacent normal coronary artery [4], and slow and turbulent flow were taken into account as well. Inter-observer variability was assessed by kappa coefficient. CAE extension was graded according to Markis Classification (Type I: diffuse ectasia of two or three vessels, Type II: diffuse disease in one vessel and localized disease in another vessel, Type III: diffuse ectasia of one vessel only and Type IV: localized or segmental ectasia) [3]. The total cohort was divided into two groups, depending on the finding of CAE or not at coronary angiography. 

### 2.3. Outcome and Follow-Up

The endpoint of our study was time to first AAE, considering it either NSTEACS, STEMI, stroke, or transitory ischemic attack (TIA). Data of follow-up were recorded by telephone interviews or by patients’ electronic health records. 

### 2.4. Ethics Statement

The research protocol complies with the Declaration of Helsinki and was approved by the ethics committee of our institution (PIC 25/2007). All patients signed informed consent documents. 

### 2.5. Biomarker and Analytical Studies

The investigators who performed the analytical studies (LB-C, MLG-C, and JLM-V) were unaware of clinical or angiographic data. Plasma concentrations of MCP-1, soluble tumor necrosis factor-like weak inducer of apoptosis (sTWEAK), and NGAL were determined in duplicate using commercially available enzyme-linked immunosorbent assay kits (BMS279/2, Bender MedSystems, Burlingame, CA, USA; BMS2006INST, Bender MedSystems, Burlingame, CA, USA; and Kit 036, BioPorto, Gentofte, Denmark, respectively) following the manufacturer’s instructions. Intra and interassay coefficients of variation were 4.6% and 5.9% for MCP-1, 6.1% and 8.1% for sTWEAK, and 5.3% and 7.9% for NGAL, respectively. Hs-CRP was assessed by latex-enhanced immunoturbidimetry (ADVIA 2400 Chemistry System; Siemens, Munich, Germany). Plasma calcidiol levels were quantified by chemiluminescent immunoassay (CLIA) on the LIAISON XL analyzer (LIAISON 25OH-Vitamin D total Assay DiaSorin, Saluggia, Italy), FGF-23 was measured by an enzyme-linked immunosorbent assay which recognizes epitopes within the carboxyl-terminal portion of FGF-23 (Human FGF-23, C-Term, ImmutopicsInc, San Clemente, CA, USA), intact parathormone (PTH) was analyzed by a second-generation automated chemiluminescent method (Elecsys 2010 platform, Roche Diagnostics, Mannheim, Germany), phosphate was determined by an enzymatic method (Integra 400 analyzer, Roche Diagnostics, Mannheim, Germany). Lipids, glucose, and creatinine levels were determined by standard methods (ADVIA 2400 Chemistry System; Siemens, Erlangen, Germany). PCSK9 was determined in duplicate by ELISA method, with anti-PCSK9 specific antibodies (ELLA kit, R & D Systems, Minneapolis, MN, USA). For lipoprotein (a) (Lp (a)), the Binding Site reagent (The Binding Site Group Ltd.; Birmingham, UK) and the SPAplus instrument from the same house were used. 

### 2.6. Statistical Analysis

Quantitative data are presented as median (interquartile range). For quantitative variables, a Student’s *t*-test was performed for those following a normal distribution, and the Mann–Whitney test was used in those not normally distributed. Qualitative variables are displayed as percentages and were compared using chi-square or Fisher’s exact test when appropriate. Analysis of normality of the continuous variables was performed with the Shapiro–Wilk test. 

All relevant clinical and analytical variables were included in a univariant binary logistic regression analysis, taking the presence of CAE as dependent variable. Thereafter, we included all variables with a level of significance *p* < 0.2 at univariate analysis in a multivariate model. This multivariate analysis was done with the backward step method, keeping only those variables with a level of significance *p* < 0.05 calculated by the likelihood ratio method. The effect of every independent predictor variable is presented as the odds ratio (OR) for CAE and its 95% confidence interval and the level of “*p*” significance, calculated by the likelihood ratio method. Furthermore, due to different prevalence of CAE between sex, we repeated the previous analyses, comparing the 23 CAE patients with 46 non-CAE patients matched for age (±2 years) and gender with a conditional logistic regression model. We used the same methodology, although with Cox proportional hazards regression, to find prognostic factors of AAE during follow-up, presenting the effect of every predictor as hazard ratio (HR) and its 95% CI. Survival-free of AAE curves were traced with the Kaplan–Maier method and groups were compared with the log-rank test.

Analyses were performed with IBM SPSS Statistics for Windows, Version 19.0 (IBM Corp. Released 2010, Armonk, NY, USA), and were considered significant when “*p*” was lower than 0.05 (two-tailed). 

## 3. Results

Two hundred and seventy patients were included in the present study. Baseline characteristics are shown in Table 1. In summary, median age was 65.0 (54.0–76.0), 66.7% were male, 20.7% had diabetes, 70% hypertension, and 46.7%/53.3% had had a STEMI/NSTEACS at index admission. 

Twenty-three patients (8.5%) were diagnosed with CAE. Kappa coefficient showed a good inter-observer agreement (k = 0.796). Attending to Markis Classification, 5 patients (21.7%) were classified as type I, 9 (39.2%) as type II, 7 (30.4%) as type III and 2 (8.7%) were classified as type IV. The most affected vessel was the right coronary artery in 17 patients (74.0%), left circumflex artery in 3 patients (13.0%), and left anterior descending artery in 3 patients (13.0%). The mean number of vessels involved by CAE was 1.91 (SD 0.85), and all the coronary dilatations were fusiform (longitudinal dimension greater than transverse diameter). 

Baseline characteristics in both groups (CAE and non-CAE) are shown in Table 1. Briefly, CAE-patients were more frequently males (91.3 vs. 64.4%, *p* = 0.004), had larger body-mass index (29.0 vs. 27.9, *p* = 0.027), and had more prevalence of dyslipidemia (82.6 vs. 47.0%, *p* = 0.001) than those without CAE. Moreover, CAE-patients had higher plasma levels of triglycerides (137.0 vs. 97.0 mg/dL, *p* = 0.005), LDL (87.0 vs. 74.0 mg/dL, *p* = 0.048), and MCP-1 (170.10 vs. 138.9 pg/mL, *p* = 0.016) than those without CAE. Plasma levels of vitamin D, FGF-23, PTH, and phosphate were similar in both groups, as well as PCSK9 and Lp (a) levels. There were no other clinical, angiographical, or analytical differences between patients with and without CAE. 

### 3.1. Predictors of CAE

At univariate logistic regression analysis, the variables that met the criterion of *p* < 0.2 were gender, history of dyslipidemia, body-mass index, glomerular flow rate, MCP-1, LDL, and triglyceride plasma levels (Table 2). At multivariate analysis, we found that the only independent predictors were gender (OR = 6.55 for male sex, 95% CI = (1.40–30.73), *p* = 0.003), history of dyslipidemia (OR = 7.39, 95% CI = (2.06–26.58), *p* < 0.001), and MCP-1 (OR = 2.25, 95% CI = (1.35–3.76) for each increase of 100 pg/mL, *p* = 0.001). Comparison of patients with CAE with 46 patients without this disorder, matched for age and gender, showed similar results, with LDL (*p* = 0.015, OR = 1.29, 95% CI = (1.03–1.61)) and MCP-1 (*p* = 0.026, OR = 2.65, 95% CI = (0.92–7.68)) being the only independent predictors. There were not significant differences in any biomarker plasma levels between patients with CAE type I or II versus those with type III and IV (not shown).

### 3.2. Prognostic Factors Associated with AAE during Follow-Up

Median follow-up was 5.35 years (range 0.09–8.99, IQR 3.88–6.65). Forty patients had a first AAE during follow-up, 8 in the CAE group (34.8%), and 32 in the control group (13.0%). The incidence of AAE was mainly driven by ACS (7 in 8) in the CAE group, whereas in the control group there was a similar incidence of coronary and neurological events (17 ACS and 15 stroke/TIA). 

**Figure 1:**
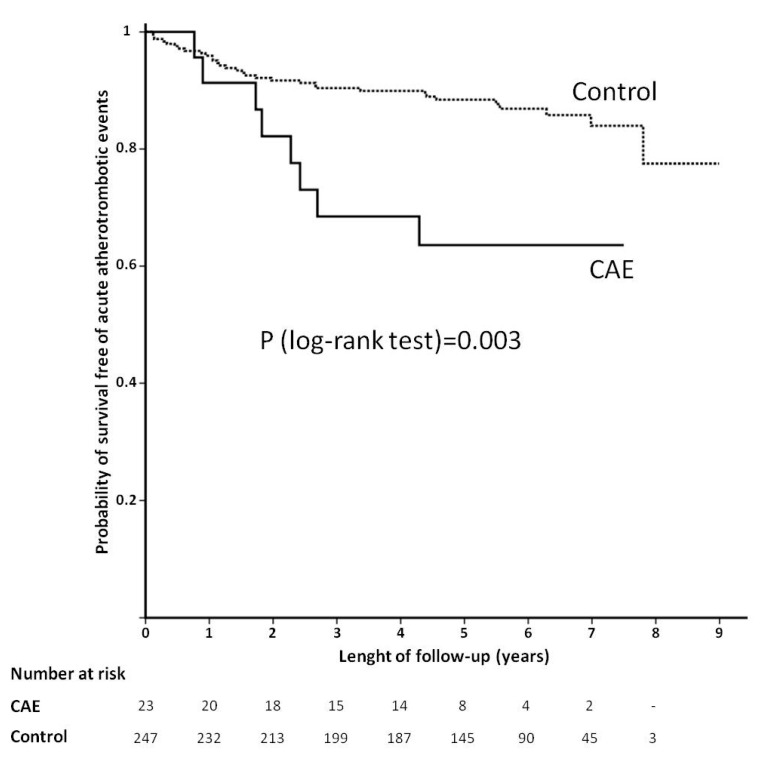
Comparison of long-term incidence of acute atherotrombotic events between patients with and without coronary artery ectasia (CAE). Survival curves are drawn with Kaplan–Meier method.

At univariate Cox regression analysis, the variables that met the criterion of *p* < 0.2 were CAE, age, hypertension, current or former smoker, body-mass index, type of ACS at index event, full revascularization, Syntax Score, hemoglobin, platelet count, triglycerides, high-density lipoprotein, calcidiol, FGF-23, PTH, MCP-1, and proton-pump inhibitors intake. The significance level and HR (95% CI) of every studied variable are shown at Table 3. After multivariate analysis, CAE was one of the strongest predictors (HR = 2.82, 95% CI = (1.29–6.15), *p* = 0.019), together with Syntax Score (HR = 1.04 for every increasing point, 95% CI = (1.01–1.06), *p* = 0.006), proton-pump inhibitors intake (HR = 0.56, 95% CI = (0.35–0.89), *p* = 0.006), and platelet count (HR = 1.68 for every increase in 100,000 units per µL, 95% CI = (1.04–2.71), *p* = 0.038). Kaplan–Meier curves of CAE and control groups are shown at Figure 1.

## 4. Discussion

### 4.1. Predictors of CAE

This is the first report to show that MCP-1 is an independent predictor of CAE. This chemokine plays a main role in the development of atherosclerosis, driving the entry of monocytes into the arterial wall and therefore contributing to the inflammatory cascade of atherosclerosis [17,18,22]. Drugs that reduce the incidence of cardiovascular events decrease MCP-1 expression in atherosclerotic lesions [23,24]. Furthermore, increased MCP-1 plasma levels have been related to acute ischemic events in patients with CAD [19,25]. Patients included in our study had proven CAD, and it is therefore presumable that most of them had the atherosclerotic form of CAE. This form could have a pathogenesis resembling that of non-ectasic CAD, with a stronger inflammatory response [1,15,26]. The higher MCP-1 levels in patients with CAE suggest that this disorder could be the result of an enhanced inflammatory status. Although other studies have found increased levels of adhesion molecules in patients with CAE [13,15], this is the first report showing an independent association MCP-1. Seemingly, this will contribute to macrophage infiltration with enzymatic degradation of extracellular matrix, weakening the arterial wall and leading to an excessive expansive remodeling of the coronary artery [1]. 

We did not find any other inflammatory biomarkers to be independent predictors of CAE. Recently, a study by Akyel et al. showed an independent relationship between plasma levels of NGAL and isolated CAE, excluding patients with CAD [14]. In our study, plasma levels of NGAL were similar between groups with and without CAE. Although this fact could reflect a low statistical power due to the small sample size, the “*p*” level in univariate analysis was quite far from statistical significance. Unlike those in Akyel’s report, all of our patients had coexisting CAD. Thus, the possibility exists that the pathogenesis of CAE in cases with coexisting CAD is not identical to that of patients where CAE presents without concomitant CAD. In recent years, sickle-cell anemia has been related to CAE, probably due to a chronic pro-inflammatory state [27]. Nevertheless, none of our patients had the disease, and its clinical relevance in our population is unlikely.

Among cholesterol metabolism components, none reached statistical significance as independent predictor. In spite of this, LDL significance was about to reach it (*p* = 0.06), which would be in accordance with other published data that showed a higher prevalence of CAE in patients with heterozygous familial hypercholesterolemia [28]. Furthermore, a positive association between isolated CAE and soluble lectin-like oxidized LDL receptor-1 levels has been described [29]. It is worth remembering that a vast majority of our patients (93%) were taking statins for secondary prevention after ACS, and therefore, current LDL level may not reflect actual lifelong burden. This could be confirmed with the fact that history of dyslipidemia, as a dichotomic variable, was one of the independent predictors in our study. High LDL levels could provoke an enhanced inflammatory response favoring CAE development. Other studies that explored differences between non-atherosclerotic CAE and controls did not find significant differences in LDL and total cholesterol levels. This could suggest a different pathogenesis between isolated and atherosclerotic CAE [14,21]. 

We also explored the potential role of PCSK9 in the genesis of CAE, but we did not find any significant relationship with its plasma levels. As far as we know, there are no published data exploring this association. It is well known that PCSK9 gain-of-function mutations cause hypercholesterolemia and premature atherosclerosis [30], and PCSK9 inhibition is an emergent target in the treatment of ischemic heart disease [31]. Although we did not find it, it cannot be ruled out that the high use of statins that are up-regulators of PCSK9 [32] could mask a possible relationship. 

There is increasing evidence of the role of abnormalities of mineral metabolism in the pathogenesis of CAD [20,33,34,35]. Demir et al. found low calcidiol—a metabolite of vitamin D—and high PTH plasma levels in a cohort of 50 Turkish patients with CAE as compared to 30 control patients [21]. Nevertheless, none of the mineral biomarkers we tested reached statistical significance. However, Demir’s population was different than ours, as they were younger, and did not have CAD. In addition, patients with chronic kidney disease, diabetes, hypertension, hypercholesterolemia, or left ventricular ejection fraction <50% were excluded in that study. On the other hand, our population displayed the typical features of subjects with CAD, with mean age around 65 years and presence of classical coronary risk factors. For these reasons, the results of the two studies are not comparable. 

In recent years, high FGF-23 plasma levels have been shown to be associated with cardiovascular disease and incidence of mortality and cardiovascular events [36,37]. In our patients we did not find any relationship between FGF-23 plasma levels and the presence of CAE. Then, although CAE and CAD may have a common pathogenesis with regard to MCP-1 and inflammation, the mechanisms of both disorders are unlikely to be identical, since abnormalities of mineral metabolism seem to play no role in CAE. 

The prevalence of CAE in our study was 8.5%, which is higher than previous works, where reported values were 0.2–5% [1,38]. We found several reasons for this discrepancy. First, there may have been a selection bias in our study, since all of our patients had undergone cardiac catheterization because of an ACS. Second, there are regional differences in CAE prevalence that could explain this variation, maybe reflecting genetic variations between different populations. Finally, the classical criterion of dilatation of a coronary segment that is 1.5 times the reference diameter may be deceptive, since very often CAE diffusely affects the entire length of a coronary vessel. In this case, it may be difficult to pinpoint the real reference diameter and, consequently, the diagnosis of CAE.

Other findings in our study were in accordance with previous publications, where male gender and history of dyslipidemia, but not diabetes, were found to be independent clinical predictors of CAE [38,39]. Similarly, the right coronary artery was the vessel most frequently affected by CAE [5].

### 4.2. CAE and Outcome

In our study, CAE was an independent predictor of AAE, despite of the fact that we have adjusted for a large number of clinical, analytical and angiographic variables, including inflammatory biomarkers. 

Until recently, data about incidence of AAE in CAE patients came from the era before current state-of-the-art therapies after ACS [3,40], showing a worse prognosis. Doi et al. studied a large retrospective cohort of 1698 patients with myocardial infarction, of whom 3% presented CAE [41]. During a median follow-up of 49 months, they reported a higher incidence of MACE (HR 3.25), cardiac death (HR 2.71), and nonfatal myocardial infarction (HR 4.92) in the CAE group, which is in agreement with our data, although the proportion of STEMI in Doi patients was significantly higher (80% vs. 47%). Another main difference was that only 22% of CAE patients and 37% of controls were taking a thienopyridine in the study of Doi et al., whereas in our study 75% of patients were taking thienopyridine given that they had a recent ACS with stent implantation in 79.6% of cases. Regarding oral anticoagulation, patients in the Doi cohort were more frequently on warfarin than those in the control group (37% vs. 13%) while its use in our study was quite lower (around 6%), due to a low prevalence of atrial fibrillation (6.3%). Data about atrial fibrillation prevalence were not reported by Doi et al.; thus, we cannot rule out this reason for oral anticoagulation in some patients. Although it has been suggested that anticoagulation might be beneficial in patients with CAE [42], this has not been tested in clinical trials, and probably, this lack of evidence provokes regional disparities. In our study, we did not find significant interaction between CAE and antiplatelets and anticoagulants use.

Finally, another relevant finding of our study was that the higher incidence of AAE in CAE group was mainly driven by ACS rather than by neurological events (88% in CAE group vs. 53% in control group), suggesting that CAE is a disorder that affects basically coronary circulation.

### 4.3. Limitations

The small size of the CAE group could limit the statistical power of our study. It would therefore be possible that other biomarkers that could play a role in the pathogenesis of CAE may have gone undetected. Due to the observational nature of our study, we cannot exclude the presence of unmeasured confounding factors that could have changed our results. On the other hand, the lack of specific genetic testing for CAE has prevented us to use a design with Mendelian randomization. Blood samples were not taken at ACS admission but rather between 6 and 12 months after the index event. Although this fact could be considered a limitation, we believe that biomarker levels could be altered if assessed during the index event. Taking into account that CAE is a chronic condition, we believe that assessing biomarker plasma levels when the patients are stable more closely reflects the chronic levels that have been present in the previous years during the phase of CAE formation. Although drugs prescribed after the index event could affect plasma levels of some of the biomarkers assessed, the treatment of patients with and without CAE was not significantly different. Regardless of the link found between MCP-1 with CAE, our study does not demonstrate a causal relationship between them; rather, these biomarkers are far from serving as diagnostic tools for CAE. Our study was initially designed to assess levels of biomarkers in a selected population with ACS, with a low burden of comorbidities, which could be interpreted as other limitation. Although Syntax Score was initially designed to guide the mode of coronary revascularization, we have used it in our work as a surrogate of CAD extent; thus, it is possible that the results would have been different using other different methods, as Gensini Score or number of vessels affected. Angiographic diagnosis accuracy of CAE might have been improved with use of intravascular ultrasound, given that this is an excellent tool for assessing luminal size and characterizing arterial wall changes. The lack of this technique in our study is other of its limitations. On the other hand, we realize that diagnosis of CAE could be challenging, especially in cases of diffuse disease, in which it would have been of interest to apply other definitions of CAE, such as Krüger et al. [43]. Finally, low sample size does not allow us to analyze the prognostic impact of CAE extent, given that we cannot provide data of outcomes in different categories of Markis classification. 

## 5. Conclusions

In conclusion, MCP-1 level is an independent predictor of CAE in CAD patients. This could mean that the two entities share a common inflammatory pathogenesis. By contrast, we did not find any relationship between CAE and PCSK9 and mineral metabolism components. Future studies are needed to investigate whether there is a causal relationship between MCP-1 plasma levels and CAE. On the other hand, CAE carries a higher incidence of AAE, and clinical trials should be developed in the future to assess the best therapy for this condition.

## Figures and Tables

**Table 1:** Clinical characteristics.

Characteristics	Global Population (*n* = 270)	Non-CAE-Group (*n* = 247)	CAE-Group (*n* = 23)	*p*-Value ^a^
Age (years)	65.0 (54.0–76.0)	66.0 (54.0–77.0)	63.0 (57.0–73.0)	0.563
Male sex (%)	66.7	64.4	91.3	**0.004**
Body mass index (kg/m^2^)	28.0 (25.6–30.9)	27.9 (25.1–30.7)	29.0 (27.0–33.6)	**0.027**
Diabetes mellitus (%)	20.7	21.1	17.4	0.794
Smoker (past or present) (%)	68.9	68.4	73.9	0.581
Hypertension (%)	70.0	69.6	73.9	0.665
Dyslipidemia (%)	50.0	47.0	82.6	**0.001**
Peripheral artery disease (%)	1.5	1.6	0.0	1.000
Cerebrovascular events (%)	5.6	6.1	0.0	0.625
Atrial fibrillation (%)	6.3	6.1	8.7	0.645
LV ejection fraction	0.60 (0.50–0.67)	0.60 (0.50–0.68)	0.57 (0.47–0.60)	0.431
STEMI (%)/NSTEACS (%)	46.7/53.3	47.0/53.0	43.5/56.5	0.748
Full revascularization (%)	70.0	71.3	56.5	0.152
SYNTAX score	14 (7–23)	14 (6–23)	19.5 (11–24.5)	0.082
ASA (%)	91.1	91.1	91.3	1.000
Clopidogrel (%)	74.8	74.9	73.9	0.917
OAC (%)	5.9	6.1	4.3	1.000
Statins (%)	93.0	93.1	91.3	0.670
ACEI (%)	69.3	70.0	60.9	0.371
ARB (%)	21.1	20.6	26.1	0.593
Beta-blockers (%)	70.7	69.6	82.6	0.171
Hemoglobin (g/dL)	14.3 (13.3–15.1)	14.3 (13.3–15.1)	14.6 (13.9–15.2)	0.402
Platelets count (per µL)	239 (191–280)	238 (191–279)	250 (191–295)	0.441
Leucocytes count (per µL)	7.2 (6.1–8.7)	7.2 (6.0–8.6)	7.8 (6.5–9.0)	0.198
% Neutrophils	59.4 (52.3–64.7)	59.4 (52.3–64.7)	57.1 (51.0–65.2)	0.746
% Lymphocytes	30.0 (24.1–36.3)	30.1 (24.1–36.3)	27.1 (24.1–36.9)	0.912
LDL cholesterol (mg/dL)	75.5 (61.0–94.0)	74.0 (61.0–93.0)	87.0 (70.0–110.0)	**0.048**
HDL cholesterol (mg/dL)	43.5 (36.0–51.0)	44.0 (36.0–52.0)	40.0 (35.0–48.0)	0.339
Triglycerides (mg/dL)	102.0 (75.8–139.3)	97.0 (72.0–137.0)	137.0 (100.0–196.0)	**0.005**
GFR (mL/min/1.73 m^2^)	75.4 (60.8–88.3)	75.2 (60.2–87.5)	80.9 (66.2–93.8)	0.212
Hs-CRP (mg/L)	1.8 (0.8–3.8)	1.7 (0.8–3.7)	2.4 (1.4–4.7)	0.154
MCP-1 (pg/mL)	142.5 (112.2–179.2)	138.9 (111.9–176.9)	170.1 (132.6–213.1)	**0.016**
NGAL (ng/mL)	165.2 (127.4–219.2)	164.2 (125.6–221.0)	167.7 (130.1–191.7)	0.982
sTWEAK (pg/mL)	198.1 (159.5–249.2)	197.8 (156.3–248.3)	206.8 (185.8–272.2)	0.254
Parathormone (pg/mL)	65.2 (49.7–83.9)	65.2 (50.2–84.3)	64.1 (48.6–77.9)	0.569
Phosphate (mg/dL)	3.3 (2.9–3.7)	3.3 (2.9–3.7)	3.3 (2.8–3.6)	0.746
FGF-23 (RU/mL)	67.6 (54.3–88.3)	68.1 (54.4–89.0)	64.0 (41.8–87.2)	0.370
Calcidiol (ng/mL)	18.4 (12.5–24.9)	18.4 (12.7–25.1)	18.4 (10.4–24.1)	0.527
PCSK-9 (ng/mL)	52.1 (42.4–64.3)	51.8 (42.0–64.3)	54.5 (46.6–66.5)	0.265
Lp (a) (mg/dL)	21.8 (7.0–48.4)	21.6 (7.0–48.2)	26.6 (12.9–67.1)	0.219

Quantitative data are presented as median (interquartile range). ACEI: Angiotensin-converting enzyme inhibitors. ARB: Angiotensin-receptor blockers. ASA: Acetylsalicylic acid. CAE: Coronary artery ectasia. FGF-23: Fibroblast growth factor-23. GFR: Glomerular Filtration Rate as estimated by CKD-EPI (Chronic Kidney Disease Epidemiology Collaboration). HDL: High-density lipoprotein. Hs-CRP: High-sensitivity C-reactive protein. LDL: Low-density lipoprotein. Lp (a): Lipoprotein (a). LV: Left ventricle. MCP-1: Monocyte chemoattractant protein-1. NGAL: Neutrophil gelatinase-associated lipocalin. NSTEACS: Non-ST-elevation acute coronary syndrome. OAC: Oral anticoagulants. PCSK-9: Proprotein convertase subtilisin/kexin type 9. STEMI: ST-elevation myocardial infarction. sTWEAK: Soluble tumor necrosis factor-like weak inducer of apoptosis. SYNTAX: Synergy between percutaneous coronary intervention with Taxus and cardiac surgery. ^a^: Comparison between CAE and non-CAE group. Bold type denotes *p*-value ≤ 0.05.

**Table 2:** Logistic regression analysis for predictors of coronary artery ectasia.

Variable	Univariate OR (95% CI)	*p* Value	Multivariate OR (95% CI)	*p* Value
Age	0.99 (0.96–1.03)	0.687	-	-
Male gender	**5.81 (1.33–25.37)**	**0.004**	**6.55 (1.40–30.73)**	**0.003**
Hypertension	1.24 (0.47–3.26)	0.665	-	-
Diabetes	0.79 (0.26–2.42)	0.673	-	-
Dyslipidemia	**5.36 (1.77–16.23)**	**0.001**	**7.39 (2.06–26.58)**	**<0.001**
Smoker	1.31 (0.50–3.46)	0.581	-	-
BMI	**1.09 (1.01–1.17)**	**0.037**	1.08 (0.97–1.20)	0.191
LVEF	0.99 (0.96–1.03)	0.686	-	-
ST-elevated ACS	0.87 (0.37–2.06)	0.748	-	-
GFR	**1.02 (0.99–1.04)**	**0.154**	1.01 (0.98–1.03)	0.581
Hemoglobin	1.11 (0.81–1.53)	0.500	-	-
Platelet count	1.00 (0.99–1.01)	0.577	-	-
Leucocytes count	1.13 (0.91–1.40)	0.288	-	-
Neutrophile (%)	1.00 (0.96–1.04)	0.892	-	-
Lymphocyte (%)	1.00 (0.95–1.05)	0.937	-	-
LDL ^1^	**2.07 (1.07–4.02)**	**0.040**	1.94 (0.87–4.34)	0.110
HDL	0.98 (0.94–1.02)	0.331	-	
Triglycerides ^1^	**1.59 (1.19–2.13)**	**0.003**	1.18 (0.84–1.67)	0.341
Calcidiol	0.96 (0.91–1.02)	0.366	-	-
Phosphate	0.84 (0.39–1.81)	0.657	-	-
FGF-23 ^2^	1.05 (0.80–1.38)	0.721	-	-
PTH	1.00 (0.98–1.01)	0.750	-	-
MCP-1 ^3^	**1.74 (1.15–2.65)**	**0.007**	**2.25 (1.35–3.76)**	**0.001**
NGAL	1.00 (0.99–1.01)	0.648	-	-
sTWEAK^3^	1.03 (0.82–1.30)	0.796	-	-
Hs-CRP	1.01 (0.98–1.04)	0.564	-	-
PCSK-9 ^4^	1.15 (0.92–1.43)	0.234	-	-
Lipoprotein (a)	1.01 (0.99–1.03)	0.205	-	-

ACS: Acute coronary syndrome. BMI: Body mass index. FGF-23: Fibroblast growth factor-23. GFR: Glomerular Filtration Rate as estimated by CKD-EPI (Chronic Kidney Disease Epidemiology Collaboration). HDL: High-density lipoprotein. Hs-CRP: High sensitivity C-reactive protein. LDL: Low-density lipoprotein. LVEF: Left ventricular ejection fraction. MCP-1: Monocyte chemoattractant protein-1. NGAL: Neutrophil gelatinase-associated lipocalin. OR: Odds ratio. PCSK-9: Proprotein convertase subtilisin/kexin type 9. PTH: Parathormone. sTWEAK: Soluble tumor necrosis factor-like weak inducer of apoptosis. ^1^ For every increase in 50 mg/dL. ^2^ For every increase in 100 RU/mL. ^3^ For every increase in 100 pg/mL. ^4^ For every increase in 100.000 ng/mL. OR of other quantitative variables refers to increase in 1 unit. Bold values denote *p* value below 0.20 at univariate analysis and below 0.05 at multivariate. *p* value was calculated by likelihood ratio method.

**Table 3:** Cox regression analysis for predictors of acute atherothrombotic events during follow-up.

Variable	Univariate HR (95% CI)	*p* Value	Multivariate HR (95% CI)	*p* Value
Age	**1.02 (0.99–1.04)**	**0.148**	0.99 (0.96–1.04)	0.958
Male gender	0.69 (0.37–1.29)	0.248	-	-
Hypertension	**2.57 (1.08–6.13)**	**0.018**	1.37 (0.53–3.53)	0.502
Diabetes	1.24 (0.59–2.60)	0.585	-	-
Dyslipidemia	1.45 (0.77–2.73)	0.244	-	-
Smoker	**0.54 (0.29–1.00)**	**0.054**	0.61 (0.28–1.33)	0.217
BMI	**1.05 (0.99–1.11)**	**0.120**	1.05 (0.98–1.11)	0.209
LVEF	0.99 (0.97–1.02)	0.691	-	-
ST-elevated ACS	**0.61 (0.32–1.18)**	**0.133**	0.67 (0.33–1-37)	0.268
PVD	0.67 (0.25–1.80)	0.477	-	-
Stroke	1.83 (0.65–5.16)	0.291	-	-
Atrial fibrillation	0.78 (0.46–1.30)	0.369	-	-
Full revascularization	**0.50 (0.27–0.93)**	**0.033**	0.65 (0.29–1.45)	0.293
Coronary artery ectasia	**3.08 (1.41–6.71)**	**0.012**	**2.82 (1.29–6.15)**	**0.019**
Syntax Score	**1.04 (1.01–1.06)**	**0.009**	**1.04 (1.01–1.06)**	**0.006**
Clopidogrel	1.05 (0.74–1.49)	0.788	-	-
VKA	0.70 (0.42–1.18)	0.224	-	-
Statins	1.29 (0.80–2.06)	0.324	-	-
Betablockers	0.84 (0.58–1.20)	0.315	-	-
ACEI/ARB	1.24 (0.82–1.86)	0.332	-	-
PPI	**0.53 (0.33–0.85)**	**0.002**	**0.56 (0.35–0.89)**	**0.006**
GFR	**0.99 (0.97–1.00)**	**0.082**	1.00 (0.97–1.02)	0.714
Hemoglobin	**0.85 (0.69–1.05)**	**0.138**	1.01 (0.77–1.33)	0.928
Platelet count ^1^	**1.73 (1.10–2.71)**	**0.021**	**1.68 (1.04–2.71)**	**0.038**
Leucocytes count	1.06 (0.90–1.24)	0.474	-	-
LDL ^2^	1.33 (0.80–2.23)	0.289	-	-
HDL	**0.98 (0.95–1.01)**	**0.132**	0.98 (0.94–1.01)	0.123
Triglycerides ^2^	**1.23 (0.98–1.54)**	**0.088**	0.93 (0.68–1.26)	0.623
Calcidiol	**0.97 (0.93–1.01)**	**0.110**	0.98 (0.94–1.03)	0.403
Phosphate	1.17 (0.65–2.08)	0.603	-	-
FGF-23 ^3^	**1.13 (0.99–1.28)**	**0.124**	1.06 (0.90–1.24)	0.510
PTH	**1.01 (0.99–1.02)**	**0.098**	1.01 (0.99–1.02)	0.426
MCP-1 ^4^	**1.25 (0.95–1.66)**	**0.168**	0.96 (0.66–1.38)	0.801
NGAL	1.00 (0.99–1.01)	0.496	-	-
Hs-CRP	0.99 (0.96–1.03)	0.496	-	-
PCSK-9 ^5^	0.92 (0.76–1.12)	0.394	-	-
Lipoprotein (a)	1.00 (0.99–1.01)	0.846	-	-

ACEI/ARB: Angiotensin-2 converting enzyme inhibitors/angiotensin-2 receptor blockers. ACS: Acute coronary syndrome. BMI: Body mass index. FGF-23: Fibroblast growth factor-23. GFR: Glomerular Filtration Rate as estimated by CKD-EPI (Chronic Kidney Disease Epidemiology Collaboration). HDL: High-density lipoprotein. HR: Hazard ratio. Hs-CRP: High sensitivity C-reactive protein. LDL: Low-density lipoprotein. LVEF: Left ventricular ejection fraction. MCP-1: Monocyte chemoattractant protein-1. NGAL: Neutrophil gelatinase-associated lipocalin. PCSK-9: Proprotein convertase subtilisin/kexin type 9. PPI: Proton pump inhibitors. PTH: Parathormone. PVD: Peripheral vascular disease. VKA: Vitamin K antagonists. ^1^ For every increase in 100,000/mm^3^. ^2^ For every increase in 50 mg/dL. ^3^ For every increase in 100 RU/mL. ^4^ For every increase in 100 pg/mL. ^5^ For every increase in 100,000 ng/mL. HR of other quantitative variables refers to increase in 1 unit. Bold values denote *p* value below 0.20 at univariate analysis and below 0.05 at multivariate. *p* value was calculated by likelihood ratio method.
